# Myomedin replicas of gp120 V3 loop glycan epitopes recognized by PGT121 and PGT126 antibodies as non-cognate antigens for stimulation of HIV-1 broadly neutralizing antibodies

**DOI:** 10.3389/fimmu.2022.1066361

**Published:** 2022-12-08

**Authors:** Veronika Daniel Lišková, Petr Kosztyu, Milan Kuchař, Jiří Černý, Shiv Bharadwaj, Hana Petroková, Eliška Vroblová, Michal Křupka, Michal Malý, Tereza Zosinčuková, Josef Šulc, Leona Rašková Kafková, Milan Raška, Petr Malý

**Affiliations:** ^1^ Laboratory of Ligand Engineering, Institute of Biotechnology of the Czech Academy of Sciences, BIOCEV Research Center, Prumyslova, Vestec, Czechia; ^2^ Department of Immunology, Palacky University Olomouc, Hnevotinska, Olomouc, Czechia; ^3^ Laboratory of Structural Bioinformatics of Proteins, Institute of Biotechnology of the Czech Academy of Sciences, BIOCEV Research Center, Prumyslova, Vestec, Czechia

**Keywords:** HIV-1, glycoprotein 120, protein scaffold, protein engineering, broadly neutralizing antibody, vaccine

## Abstract

**Introduction:**

Imprinting broadly neutralizing antibody (bNAb) paratopes by shape complementary protein mimotopes represents a potential alternative for developing vaccine immunogens. This approach, designated as a Non-Cognate Ligand Strategy (NCLS), has recently been used for the identification of protein variants mimicking CD4 binding region epitope or membrane proximal external region (MPER) epitope of HIV-1 envelope (Env) glycoprotein. However, the potential of small binding proteins to mimic viral glycan-containing epitopes has not yet been verified.

**Methods:**

In this work, we employed a highly complex combinatorial Myomedin scaffold library to identify variants recognizing paratopes of super candidate bNAbs, PGT121 and PGT126, specific for HIV-1 V3 loop epitopes.

**Results:**

In the collection of Myomedins called MLD variants targeted to PGT121, three candidates competed with gp120 for binding to this bNAb in ELISA, thus suggesting an overlapping binding site and epitope-mimicking potential. Myomedins targeted to PGT126 designated MLB also provided variants that competed with gp120. Immunization of mice with MLB or MLD binders resulted in the production of anti-gp120 and -Env serum antibodies. Mouse hyper-immune sera elicited with MLB036, MLB041, MLB049, and MLD108 moderately neutralized 8-to-10 of 22 tested HIV-1-pseudotyped viruses of A, B, and C clades *in vitro*.

**Discussion:**

Our data demonstrate that Myomedin-derived variants can mimic particular V3 glycan epitopes of prominent anti-HIV-1 bNAbs, ascertain the potential of particular glycans controlling neutralizing sensitivity of individual HIV-1 pseudoviruses, and represent promising prophylactic candidates for HIV-1 vaccine development.

## Introduction

Human immunodeficiency virus type 1 (HIV-1) is one of the potential pathogenic exogenous retroviruses, which is quickly evolving into new subtypes driven by the high frequency of mutation, recombination, and replication (1). Notably, mutations that assist the virus in escaping from host immune surveillance are positively selected as the dominant variance for integration in the viral population (2). Therefore, HIV-1 has been genetically evolved and typically characterized into four main groups, named M (main), O (outlier), N (non-M/non-O), and P (3–5). Furthermore, HIV-1 group M evolved into 11 clades (A–H, J, K, and U, where clades A and F possess subclades marked as A1/A2/A5/A6 and F1/F2, respectively). Recently, 132 circulating recombinant forms (CRFs) have been recognized (last accessed November 12, 2022, see https://www.hiv.lanl.gov/content/sequence/HIV/CRFs/CRFs.html) along with multiple HIV-1 unique recombinant forms (URFs). Moreover, a positive correlation was demonstrated between the rates of evolution in HIV-1 and disease progression (6). Hence, the prevalence of high genetic diversity in HIV-1 has been suggested as a major obstacle for the development of an efficacious prophylactic vaccine and contributing to the emergence of resistance against the available therapeutics.

The HIV-1 envelop (Env) protein, a trimer of gp41/gp120 heterodimers, exhibits 30 ± 3 potential N-linked glycosylation sites (PNGSs) per protomer (7), and it has been elucidated for its essential function in the HIV biology (8). The N-terminal subunit, gp120 protein, comprises five variable regions (V1-V5) infused with five conserved regions (C1-C5) (9). The gp120 protein exhibits a high degree of variability in different genotypes of HIV-1, but the positions of PNGS are mostly conserved among the diverse clades and isolates (10). Though the host immune surveillance can distinguish the HIV-1 targets determinants dispersed across the viral genome, the availability of HIV-1 gp120 protein on the surface makes it an ideal antigen for eliciting humoral responses against HIV-1 that typically initiated within a month after measurable plasma virus loads in the infected individuals (11). As the neutralizing antibodies (NAbs) are produced only after several months of viral infection (12), such an event results in an escape route for the coexistent viruses by generating virions relatively resistant to autologous HIV-1 NAbs (13).

Among the demarcated five variable loops in HIV-1 gp120, the V3 loop has been demonstrated as a conserved region with amino acid variation limited to ~20% of the loop’s residues (14), essentially affecting the binding with co-receptor expressed on host cells (15). Interestingly, the HIV-1 gp120/V3 loop is characterized as highly immunogenic, while V3 loop deletion has been demonstrated to abrogate viral infectivity (16). In addition, anti-V3 loop monoclonal antibodies have been isolated from the sera of HIV-1 infected patients (17). Moreover, the structural analysis of the V3 peptide crystallized with different V3-specific antibodies isolated from HIV-infected individuals demarcated three separate sites in the gp120/V3 loop: (i) the base (residues 296-299 and 327-331), (ii) the stem (residues 300-303 and 321-326) and (iii) the crown (residues 304-320) (18). Despite variation in viral strains, conserved glycan cluster centered on N332/N334 is noted for PNGSs at N295, N301, N386 and N392 (19). The abundant density of these glycans, which restricts the processing in this region and results in highly conserved amino acid stretch across all viral Clades, give rise to its “intrinsic” nature (20, 21). Notably, the N332 supersite in the base of V3 loop is considered as the vulnerable region for full antibody-facilitated virus neutralization and is attracting attention in the ongoing effective vaccine design and development against HIV-1. For instance, glycan supersite targeting antibodies include the prototype 2G12 antibody (22) and twelve antibodies, named PGT121-137, isolated from three elite neutralizers (23).

Recently, we have demonstrated that small binding proteins obtained from the scaffold of the albumin binding domain (ABD) specifically recognize the paratope of VRC-01 bNAb and compete with gp120 protein for binding to VRC-01 bNAb. These proteins, called VRA binders, mimic natural epitopes in the CD4 binding region of gp120 protein, and when used as immunogens, they stimulate the production of anti-gp120 antibodies. Hyperimmune mouse sera show the neutralization of HIV-1 pseudoviruses of Clades B and C *in vitro* (24). This approach, designated as the “Non-cognate ligand strategy” (NCLS) (25), was used to develop Myomedin scaffold-derived MLA proteins mimicking epitope of 10E8 bNAb in the membrane-proximal external region (MPER) of gp41 protein (26). Elicited anti-Myomedin mouse sera antibodies moderately neutralized up to 12 of 22 tested HIV-1 pseudoviruses of Clades A, B and C (26). As glycan epitopes located in a high-mannose patch of gp120 protein represent prominent targets of several HIV-1 bNAbs, we employed the NCLS approach to select protein imprints of glycan supersite targeting paratopes of PGT121 and PGT126 bNAbs using a highly complex Myomedin scaffold library and identified several protein variants with shape and electrostatic complementarity that mimic cognate glycan epitopes.

## Materials and methods

### Myomedin combinatorial library assembly

The combinatorial library of Myomedin variants was designed and assembled using a series of polymerase chain reactions (PCR) by Phusion High-Fidelity DNA Polymerase (NEB, Massachusetts, USA), as reported earlier (26).

### Antibodies collection

Broadly neutralizing anti-HIV-1 gp120 monoclonal antibodies PGT121 (cat# ARP-12343) and PGT126 (cat# ARP-12344) were acquired from the NIH AIDS Reagent Program (Division of AIDS, NIAID, National Institute of Health, Germantown, MD, https://www.hivreagentprogram.org). Both the PGT121 and PGT126 IgG1 λ type were employed as target proteins for ribosome display (RD) selection and in the enzyme-linked immunosorbent assay (ELISA) applications. Human IgG1 with λ light chain purchased from Sigma-Aldrich, St. Louis, MO, was utilized as an isotype control in the preselection of the library during RD as well as an isotype control in ELISA. All the other secondary antibodies of molecular grade were used in this study.

### Ribosome display selection

The combinatorial Myomedin variant library was used for three rounds of *in vitro* transcription/translation and RD selection, as reported earlier (26). Then, 96-well PolySorp plates (Thermo Scientific™ Clear Flat-Bottom Immuno Nonsterile 96-Well Plates) were immobilized with a constant concentration (25 µg ml^-1^) of human IgG1 λ type and varied concentrations (first round: 50 µg ml^-1^, second round: 50 µg ml^-1^, third round: 20 µg ml^-1^) of PGT121 or PGT126 bNAbs in 100 mM bicarbonate/carbonate buffer (pH 9.6) were used to conduct the preselection and adjust the stringency during each round of RD selection, respectively. To increase the stringency during each round of RD selection, different concentrations of TWEEN 20 in phosphate buffered (pH 7.2), i.e., 0.05% Tween/5 times, 0.05% Tween/10 times, and 0.25% Tween/10 times were used in the first, second, and third cycle, respectively. Following, the complementary DNA (cDNA) collected from the third round of RD selection was amplified into double-stranded DNA using PCR with primers JOIN-F(CTATAGGGAGACCACAACGGTTTCCCTCTAGAAATAATTTTGTTTAACTTTAAGAAGGAGATATACATATGAAAAGCGAGCTGGCCG) and JOIN-R (GAACCGACCGCGGATCCACCCTGTTTACGAATCCATTCTT), and the resulting product was further amplified with primers His-Myo-F (CAGTCCATGGGCAGCAGCCATCATCATCATCATCACAGCAGCGGCAAAAGCGAGCTGGCCG) and JOIN-R to insert cloning sites, as reported earlier (26). Finally, the amplified combinatorial Myomedin library DNA was digested using NcoI/BamHI restriction enzymes and inserted as a fusion with the V5-tag sequence in a pET-28b cloning vector resulting in the cloned cDNA plasmid library that was used to transform *E. coli* XL1 blue host cells. The sequences of particular bacterial clones expressing complete Myomedin variants were considered for further analysis.

### Myomedin variants production

The selected Myomedin proteins (~16 kDa) containing hexahistidine (His6)-tag at the N-terminus and V5-tag at the C-terminus (His6-Myomedin-V5) were expressed in *E. coli* BL21 (DE3) cells cultured in Luria-Bertani (LB) broth with kanamycin (60 µg ml^-1^). Briefly, the bacteria culture was incubated at 37°C to reach the optical density (OD_600_) ~0.8 under continuous shaking conditions (230 rpm), followed by adding 1 mM IPTG for protein production at 32°C for the next 4 h on shaking conditions. Later, the cells were harvested by centrifugation (6000 ×g, 4°C) and suspended in TN buffer (50 mM Tris, 150 mM NaCl, pH 8.0), disrupted using a sonicator (Misonix Sonicator^®^ 3000 Ultrasonic Cell Disruptor), and the supernatant containing soluble protein was collected by centrifugation (20 min, 40 000×g, 4°C). The cell lysates were then analysed and used for protein purification on the Ni-NTA agarose column. Myomedin variants containing C-terminal Avi-tag sequence consensus were also produced (His6-Myomedin-Avi) in *E. coli* BL21 (DE3) BirA strain cultivated in LB broth with 50 µM d-biotin (prepared from 5 mM solution in 10 mM Bicine buffer, pH 8.3), kanamycin (60 µg ml^-1^), and chloramphenicol (30 µg ml^-1^).

### ELISA and competition ELISA

ELISA was used to screen the Myomedin variants binding with the selected bNAbs compared to isotype control (human IgG1 λ type). Herein, 5 µg ml^-1^ of PGT121 IgG1, PGT126 IgG1, or IgG1 λ diluted in 100 mM bicarbonate/carbonate solution, pH 9.6, were immobilized in 96-well PolySorp plates (Thermo Scientific™ Clear Flat-Bottom Immuno Nonsterile 96-Well Plates) at 4°C overnight. In the following steps, the plates were washed three times with PBS (pH 7.4) containing 0.05% Tween 20 solution (PBST), and wells were blocked with PBST with 1% BSA (PBSTB) for 2 h at room temperature (RT). Then, the cell lysate samples or purified Myomedin proteins diluted in PBSTB were incubated in a 96-well plate for 1 h and later detected for binding with coated antibody using anti-V5-tag–HRP (Abcam, Cambridge, UK) in PBSTB (1:10 000 dilution). For competition assay, multimerizing HIV-1 consensus B gp120 protein prepared and characterized in detail previously (27–29) was serially diluted in PBSTB and used as a competitor of Myomedin variants (His-Myo-Flag or His-Myo-Avi) at a constant concentration (~10 µg ml^-1^) and incubated in the 96-well plate for 1 h at RT. Then, the Myomedin variants were detected by anti-Flag tag antibody (1:8 000 dilutions, HRP Anti-DDDDK tag antibody [M2], Abcam, Cambridge, UK) or by streptavidin–HRP conjugate (1:10 000 dilution, Pierce™ High Sensitivity Streptavidin-HRP, Thermo Fisher Scientific, Waltham, MA USA) in the case of *in vivo* biotinylated Avi-tagged variants. In both ELISA setups, results were observed as the reaction of HRP with TMB (3, 3’, 5, 5’-tetramethylbenzidine)-Complete 2 substrate (TestLine Clinical Diagnostics s.r.o., Brno, Czech Republic), which was stopped by 2 M sulfuric acid. For binding or competition assay, the absorbance at 450 nm was measured using the Multi-Mode ELISA Reader (BioTek Synergy™ HTX system).

### Modeling of MLD-PGT121 and MLB-PGT126 interactions

The structure of Myomedin-derived MLD proteins was modeled using the MODELLER 9v14 suite of programs (30) based on the parental non-randomized myomesin-1 domain 10 structure (PDB ID 3rbs (31), residues 1246-1358). The structure of the PGT121 bNAb was obtained from the 4fqc (32) crystal structure. A model of the PGT121 in complex with the HIV-1 gp120 was built by combining information from the 4fqc and 5cez (33) structures. A homology model of the PGT126 in complex with the HIV-1 gp120 was prepared with MODELLER using chains G and J of the 5aco (34) PGT128/Env complex cryo-EM structure as the template. The flexible side chain protein-protein global docking was performed using a local copy of the ClusPro server (35, 36) docking the MLD variants (as ligands) to the interacting domains of PGT121 using chain H residues 1-112 and chain L residues 10-107 of the 4fqc structure, or residues 1-110 from chain H and chain L residues 6-109 of the 5cez PGT121-precursor structure (as receptors). The MLB variants were docked to interacting domains of the PGT126 model as well as to the corresponding parts of the parent PGT128 structure 5aco (chain G residues 2-111 and chain J residues 2-106). Throughout the paper, the antibody residues are numbered according to their template structures, 4fqc for PGT121 and 5aco for the PGT126 model. The identification of residues involved in predicted Myomedin/antibody binding interactions, measurement of their inter-residue distances, and assembly of accompanying molecular graphics was performed in PyMOL version 2.4.0 (PyMOL Molecular Graphics System, Version 2.4 Schrödinger, LLC).

### Immunization of experimental mice

Experimental female BALB/c mice of weight 18-22 g and 6-8 weeks old (AnLab, Brno, Czech Republic), housed following ARRIVE guidelines (37), were used as recipients of selected Myomedin proteins as immunogens. A constant concentration of 10 µg in 50 µL of PBS of individual Myomedin variants was mixed with 50 µl Freund´s adjuvant (Sigma Aldrich, St. Louis, MO, USA) and injected into mice by intradermal route (five mice per group) per each immunization round. Afterwards, sera were collected before each round of immunization to monitor the HIV-1 Env neutralizing antibodies production. The Ethics Committee of the Faculty of Medicine and Dentistry (Palacky University, Olomouc, Czech Republic) and the Ministry of Education, Youth and Sports, Czech Republic (MSMT-9487/2019-3) authorized the immunization experiment on mice.

### Determination of HIV-1 Env-specific antibodies in mouse sera

The binding of serum antibodies targeting HIV-1 Env was determined using multimerizing HIV-1 consensus B gp120 protein (27–29) and a representative pseudovirus of Clades A, B and C in ELISA. Pseudoviruses were produced in HEK293 cells by co-transfection with plasmid DNA encoding viral Env (4 µg) and plasmid coding pSG3deltaEnv (8 µg) diluted in 752 µl of DMEM and mixed with 48 µl of transfection reagent FuGene6 (Promega, Madison, WI, USA). Later, the pseudoviruses were harvested after two days of incubation, filtered, and stored at -80°C. Pseudoviruses were ultracentrifuged (50000×g for 3 h at 4°C) and resuspended in PBS (pH 7.4). In the following steps, MaxiSorp 96-well plates (NUNC, Roskilde, Denmark) were coated with gp120 or pseudoviruses diluted in PBS overnight at 4°C. Then, plates were washed three times with PBS and blocked with PBSTB solution for 3 h at RT. The serially diluted mouse sera in blocking buffer were added to the wells, followed by overnight incubation at 4°C and washed with PBST solution. The antibodies targeting gp120 or particular pseudoviruses were measured with rabbit anti-mouse IgG secondary antibody conjugated with horseradish peroxidase diluted in a blocking buffer for 3 h at RT. Finally, the plates were washed with PBS solution, and the results were observed as an enzymatic reaction of HRP with O-phenylenediamine-hydrogen peroxide substrate. The enzyme reaction was stopped by adding 1M sulphuric acid, and absorbance at 492 nm was quantified using the ELISA reader.

### Competition of mouse sera with bNAbs “solid phase” setup

ELISA plates coated with 50 ng/well of multimerizing HIV-1 consensus B gp120 (27–29) protein were incubated with bNAbs (PGT121 or PGT126) serially diluted in blocking buffer containing mouse hyperimmune serum (1:200 dilution) for 3 h at RT. After that, plates were washed three times and captured serum antibodies were detected by HRP-conjugated rabbit anti-mouse IgG secondary antibody (1:2000 dilution in blocking buffer). The results were obtained as described in the previous section.

### Competition of mouse sera with bNAbs “in solution” setup

In individual Epp tubes PGT121 or PGT126 antibodies were serially diluted in blocking buffer, mixed with 100 ng of gp120 and 2 µl of individual serum in total volume of 200 µl, incubated at room temperature for 2 hours and then applied onto gp120-coated and blocked ELISA plates. After overnight incubation at 4°C, plates were washed, and bound mouse antibodies were detected as in previous section.

### Virus neutralization assay

The assay was performed following the protocol described previously (24, 38) utilizing pseudoviruses of Clades A, B, C, D, and AE secreted from the HEK293 cell line as discussed above. Likewise, Murine leukemia virus (MULV)-pseudovirus, containing the Env of MULV in the same vector as all HIV-1-pseudotyped viruses (pSG3ΔEnv), was produced under similar conditions and used as a negative control. After heat-deactivation, sera were serially diluted and incubated with pseudoviruses (150,000 RLU in 50 µl) at 37°C for 90 min. For monitoring the pseudovirus infection, the TZM-bl cell reporter system based on the stable expression of CD4 and two co-receptors, CCR5 and CXCR4, was used as described earlier (26).

### Statistics

Analysis of variance (ANOVA), Kruskal-Wallis test and Dunn´s post-test were used to determine the statistical significance of differences between groups by statistical packages SPSSv.21 GraphPad Prism 5 Software (GraphPad Software Inc., San Diego, CA, USA) or (IBM Corp., Armonk, NY, USA).

## Results

### Identification of Myomedin variants binding to HIV-1 bNAbs

**Figure 1:**
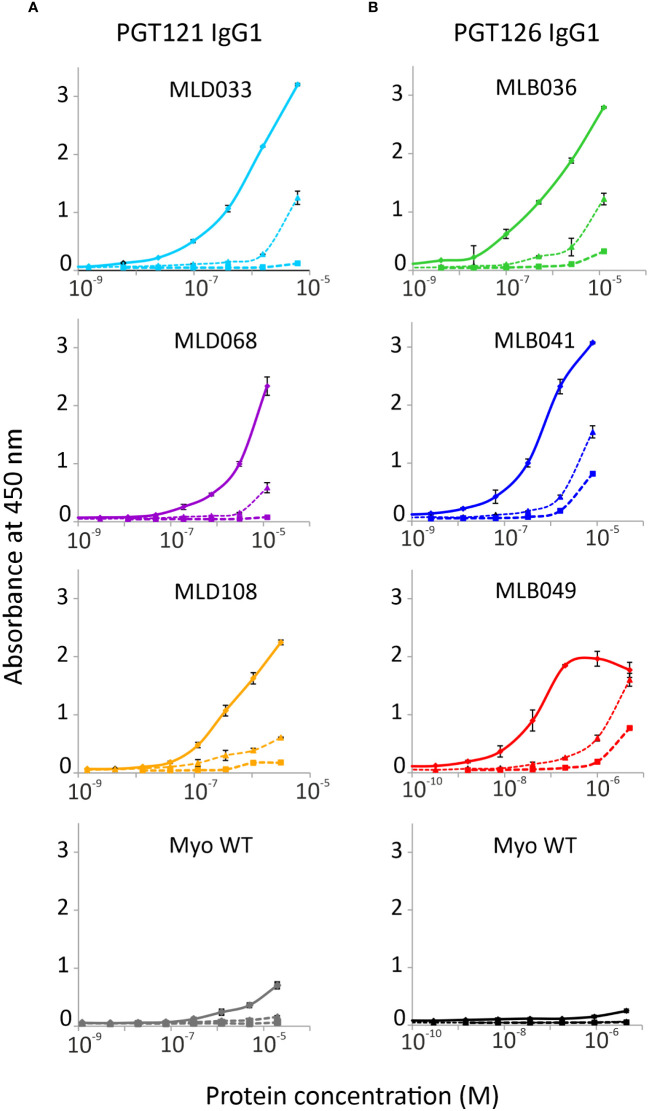
Binding of **(A)** MLD and **(B)** MLB protein variants to PGT121 and PGT126 bNAbs, respectively. The selected purified MLD proteins 033, 068 and 108, and MLB Myomedins 036, 041 and 049 with N-terminal His6-tag and C-terminal V5-tag were assayed in ELISA. Myomedin wild type (Myo WT) was added as a negative control. The binding of Myomedins to immobilized PGT121 and PGT126 bNAbs (solid line), IgG1λ isotype (dashed line with triangles) and BSA (dashed line with square) was detected by anti-V5 Ab-HRP conjugate. Each point is shown as the mean value with standard deviation.

**Figure 2:**
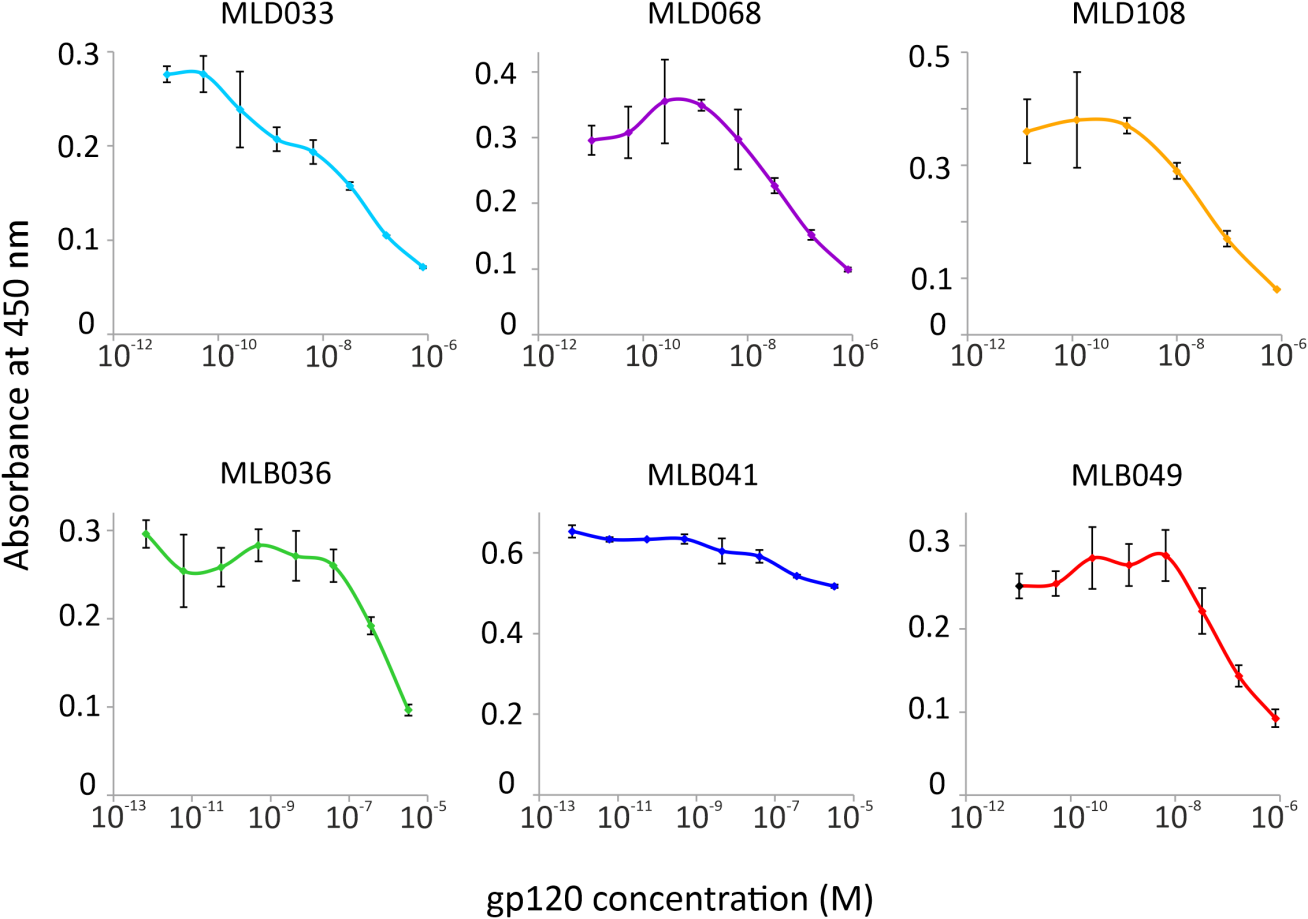
Competition of MLD and MLB protein variants with HIV-1 gp120 for binding to PGT121 or PGT126 bNAb in ELISA, respectively. The purified Myomedin variants MLB036, MLB041, MLD033, MLD108 (His6-Myo-Flag) and *in vivo* biotinylated variants MLD068 and MLD049 (His6-Myo-Avi) were assayed at a constant concentration 0.5 µM with increasing concentration of gp120, and their binding to PGT121 or PGT126 bNAbs was visualized by the anti-Flag M2 Ab-HRP conjugate or by streptavidin-HRP. Each experiment was performed in triplicates and values are given as an average with standard deviation.

To verify the ability of Myomedin scaffold proteins to mimic glycan epitopes as a critical part of high-mannose patches on gp120 recognized by HIV-1 broadly neutralizing antibodies, two bNAbs, i.e., PGT121 and PGT126, were chosen as molecular targets. Monoclonal antibody PGT121 belongs to a group of super-candidate anti-HIV-1 neutralizing antibodies due to its potential to neutralize a wide portfolio of HIV viruses of several clades with high efficacy. Independently, PGT126 as a suitable representative of the PGT128 sub-family was selected as another V3 loop glycan targeting bNAb with high coverage of HIV-1 variants. Both antibodies were used as targets for the selection of Myomedin proteins using 3-round RD. Large-scale screening of bacterial lysates of individual Myomedin variants preferentially binding to the particular immobilized bNAb resulted in the identification of three variants of anti-PGT121 proteins called MLD033, MLD068 and MLD108, and three variants of anti-PGT126 Myomedins called MLB036, MLB041 and MLB049 (**Figure 1** and **Supplementary Figure S1**). Further analysis demonstrated that MLD proteins (MLD033, MLD068 and MLD108) and MLB proteins (MLB036, MLB041 and MLB049) competed with gp120 for binding to the PGT121 and PGT126, respectively (**Figure 2**), suggesting an overlapping binding site and epitope-mimicking potential. Protein sequences of MLB and MLD variants are shown in **Table 1**.

**Table 1:** Sequence comparison for the selected MLB/MLD protein variants.

Myomedin variant	Randomized amino acid positions											
	L1				L2			L3				
	21	22	23	24	50	51	52	76	77	78	79	80
**Myo WT**	E	K	L	S	R	N	T	D	G	K	A	T
**MLB036**	M	W	R	N	R	N	T	W	M	T	Q	T
**MLB041**	I	M	M	E	D	M	R	I	V	T	P	L
**MLB049**	K	H	Q	L	W	L	W	I	V	T	P	L
**MLD033**	H	W	Q	F	Q	G	E	P	Q	L	W	L
**MLD068**	Y	A	G	N	V	Q	Y	E	P	I	F	L
**MLD108**	H	G	Q	W	V	S	L	Q	T	A	T	Y

Myomedins containing 12 randomized amino acid position in the variable loop (L1-L3) regions are shown with parental non-randomized wild type Myomedin (Myo WT).

### Modeling of MLB and MLD interactions with PGT126 and PGT121 monoclonal antibodies

**Figure 3:**
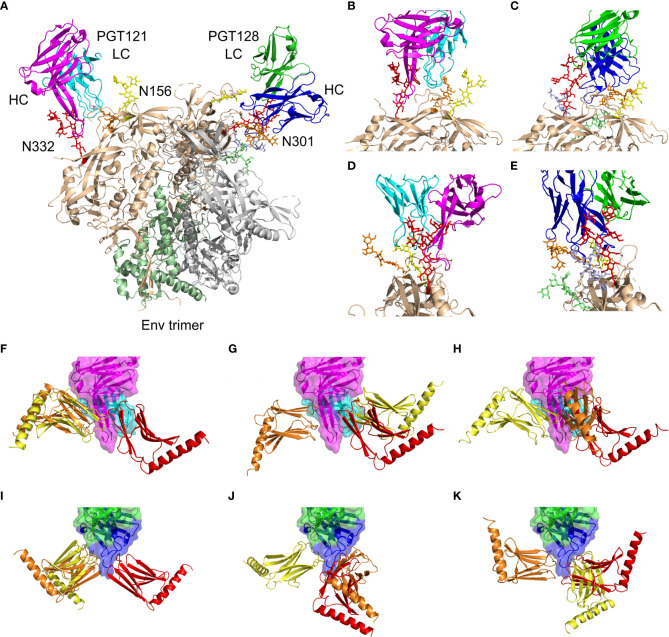
Summary of PGT121 and PGT126 interaction modes with the HIV-1 Env trimer and with reported Myomedin variants. **(A)** A collage of PGT121 precursor [5cez (33)] heavy chain (magenta) and the light chain (cyan) and the PGT128 [5aco (34)] heavy chain (blue) and the light chain (green) in complex with Env trimer showing gp41 (palegreen), gp120 (wheat), and the interacting glycans at N332 (red), N301 (orange), N156 (yellow), N262 (lime), and N295 (lightblue). **(B, D)** Enlarged and rotated view of the PGT121 interaction with gp120 (wheat) and glycans at N332 (red), N301 (orange), and N156 (yellow). **(C, E)** Enlarged and rotated view of the PGT128 interaction with gp120 (wheat) and glycans at N332 (red), N301 (orange), N156 (yellow), N262 (lime), and N295 (lightblue). **(F–K)** The predicted binding modes of Myomedin variants targeting PGT121 [4fqc (32)] heavy chain (magenta) and the light chain (cyan) are shown for **(F)** MLD033, **(G)** MLD068, and **(H)** MLD108. Similarly, the PGT126 [homology model built from PGT128 template, 5aco (34)] heavy chain (blue) and the light chain (green) binding modes are shown for **(I)** MLB036, **(J)** MLB041, and **(K)** MLB049. The typical binding modes color-coded by predicted binding probability in descending order from red through orange to yellow. A more detailed view is available in **Supplementary Figures** and PyMOL session at 10.5281/zenodo.6913156.

We analyzed interactions of PGT121-124 and PGT125-128 subfamilies based on available structural data. The first subfamily can be represented by the selection of PDB structures containing Env/PGT121-precursor complex 5cez (33), glycan-bound PGT121 4fqc (32), Env/PGT122 complex 4nco (9), and gp120-bound PGT124 4r2g (39). The second subfamily can be represented by glycan-bound PGT127 3twc (39) and 3tv3 (39) PGT128 structures, and the Env/PGT128 complex structure 5aco (34). The combined structural data revealed localized direct protein-protein interactions surrounded by a number of interacting glycans at N137, N156, N262, N295, N301, and N332. To assess the binding contributions, we measured distances between involved protein residues and glycan moieties identifying patches of closely interacting residues. The color-coded summary of involved residues and glycans N137, N156, N301, and N332 is available in **Supplementary Figures 2**, **3**. The Env/antibody interaction modes with selected glycan moieties are summarized in **Figures 3A–E**, and complete structural data are available as Supplementary PyMOL sessions at 10.5281/zenodo.6913156.

To verify the most probable antibody binding areas for all selected MLB and MLD binders, we performed a flexible side chain global docking of corresponding proteins using ClusPro. Docking results are presented in **Figures 3F–K**, revealing that MLB and MLD binders employ mostly interfaces corresponding to N137, N301, and N332 glycans.

### MLB and MLD proteins elicit HIV-1 anti-Env serum antibodies

**Figure 4:**
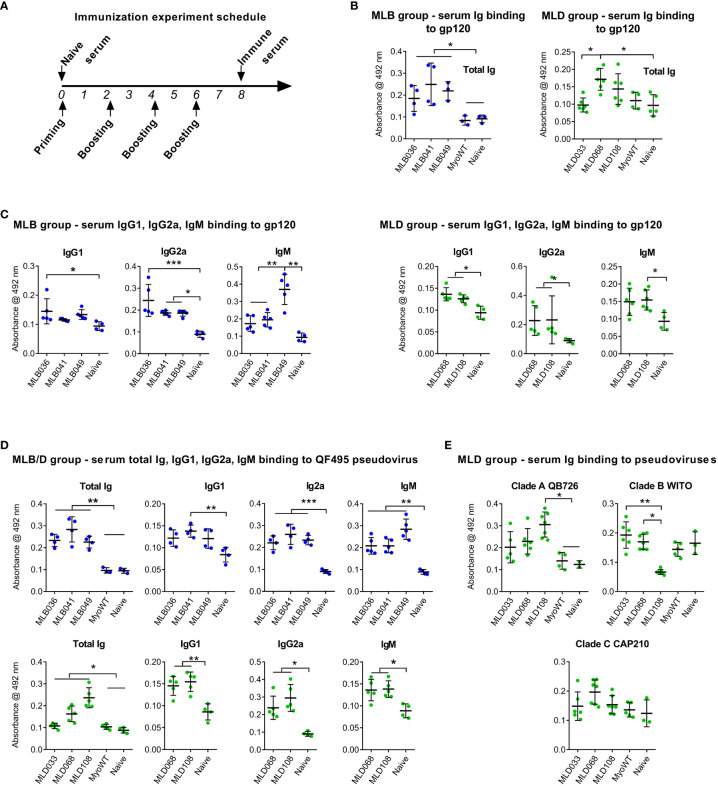
MLB and MLD proteins elicited anti-HIV-1 Env serum antibodies. Experimental animals were immunized four times with particular Myomedin variants by intradermal route **(A)**, sera were collected and tested for binding to multimerizing HIV-1 gp120 clade B **(B, C)** and selected pseudoviruses **(D, E)** immobilized on 96-well plates. Antibody titers of IgG **(B, E)**, IgG1, IgG2a and IgM **(C, D)** isotypes were measured in ELISA. ANOVA Kruskal-Wallis test with Dunn’s post-test (* P < 0.05, ** p < 0.01, *** p < 0.005) was used for statistical significance.

Myomedin variants, MLB036, MLB041, MLB049, MLD033, MLD068, MLD108, and parental MyoWT, were injected as antigens into mice by intradermal route with Freund’s adjuvant by four series of immunizations as presented in **Figure 4A**. The ability of identified MLB and MLD proteins to induce Env-specific anti-HIV-1 serum antibodies was determined by ELISA (**Figure 4B**) with the multimerizing version of consensus B gp120 glycoprotein as characterized earlier (27, 28). The most dominant anti-Env-specific immune response was stimulated by MLB036, MLB041, MLB049, MLD068, and MLD108 proteins (**Figure 4B**). In the case of MyoWT protein, the particular sera did not produce any detectable anti-HIV-1 Env serum response in comparison to the sera of naive mice. Serum antibody response specific for gp120 in total IgG isotype, IgG1, IgG2a, and IgM isotypes is shown in **Figure 4** **C**. MLB induced well-detectable gp120-specific responses in IgG2a and IgM for all tested MLB variants, and variant MLB036 also induced IgG1 antibodies. MLD Myomedins induced well-detectable gp120-specific responses in IgG2a and IgG1 for all tested MLD variants and variant MLD108 also induced IgM antibodies (**Figure 4C**). In addition to recombinant multimerizing consensus B gp120, we tested the reactivity with Clade B pseudovirus QF495 (**Figure 4D**) coated on the ELISA plate. The experiment identified significant binding of all MLB- and MLD-immunized murine sera to QF495 in total IgG, IgG2a and IgM isotypes and for MLB041-, MLD068-, and MLD108-immunized groups in IgG2a isotype (**Figure 4D**). To test possible differences in the binding of total serum immunoglobin (Ig) to various glycovariants of HIV-1 Env, the reactivity of MLD-immunized mouse sera with selected pseudoviruses of clade A, B, and C was tested in the ELISA (**Figure 4E**). The most prominent differences were detected for MLD108 particularly to variant Clade B WITO, which was not recognized by sera of this group.

**Figure 5:**
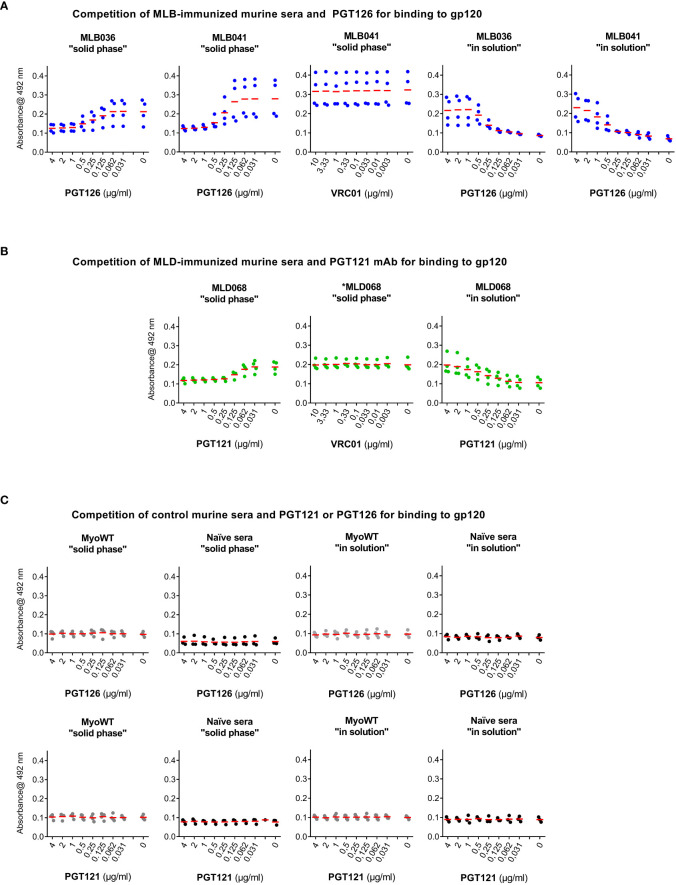
PGT126 and PGT121 compete with sera from MLB- or MLD-immunized mice for binding to HIV-1 multimeric gp120. Binding of sera from **(A)** MLB036-, MLB041- or **(B)** MLD068-immunized mice to recombinant multimerizing HIV-1 consensus B gp120 protein was inhibited by increasing concentrations of PGT126 or PGT121. As a control, irrelevant VRC01 anti-HIV-1 mAb was used. VRC01 mAb reactivity with used multimerizing consensus B gp120 was reported earlier (29). The competition was performed either by addition of the hyperimmune serum to the gp120-coated well together with individual mAb (at indicated dilution), designated as a “solid phase” competition or the serum was preincubated with gp120 and individually diluted mAb and after incubation the mixture was added to gp120-coated well, designated as “in solution” competition. **(C)** Naïve sera or sera from MyoWT-immunized mice were used as controls tested both in “solid phase” and “in solution” conditions. All tested mouse sera were diluted (1:200) and their binding was visualized using HRP-conjugated rabbit anti-mouse IgG mAb. The mean values are indicated by horizontal lines.

To verify the anti-gp120 reactivity of hyperimmune sera, a competition assay with sera from MLB036-, MLB041-, and MLD068-immunized mice was performed. The binding of mouse sera to immobilized multimerizing consensus B-type gp120 competed with serially diluted PGT126 or PGT121 as a specific target for MLB and MLD, respectively (**Figures 5A, B**). PGT126 and PGT121 in concentrations 2 µg ml^-1^ and 0.25 µg ml^-1^ inhibited the reactivity of MLB- and MLD-elicited hyperimmune sera. When irrelevant VRC01 bNAb was used, no competition was observed even at the concentration of 10 µg ml^-1^. Naive sera and MyoWT-immunized mouse sera did not titrate with PGT126 and PGT121 (**Figure 5C**).

### MLD and MLB proteins elicit hyperimmune mouse sera neutralizing a set of HIV-1 pseudoviruses

**Figure 6:**
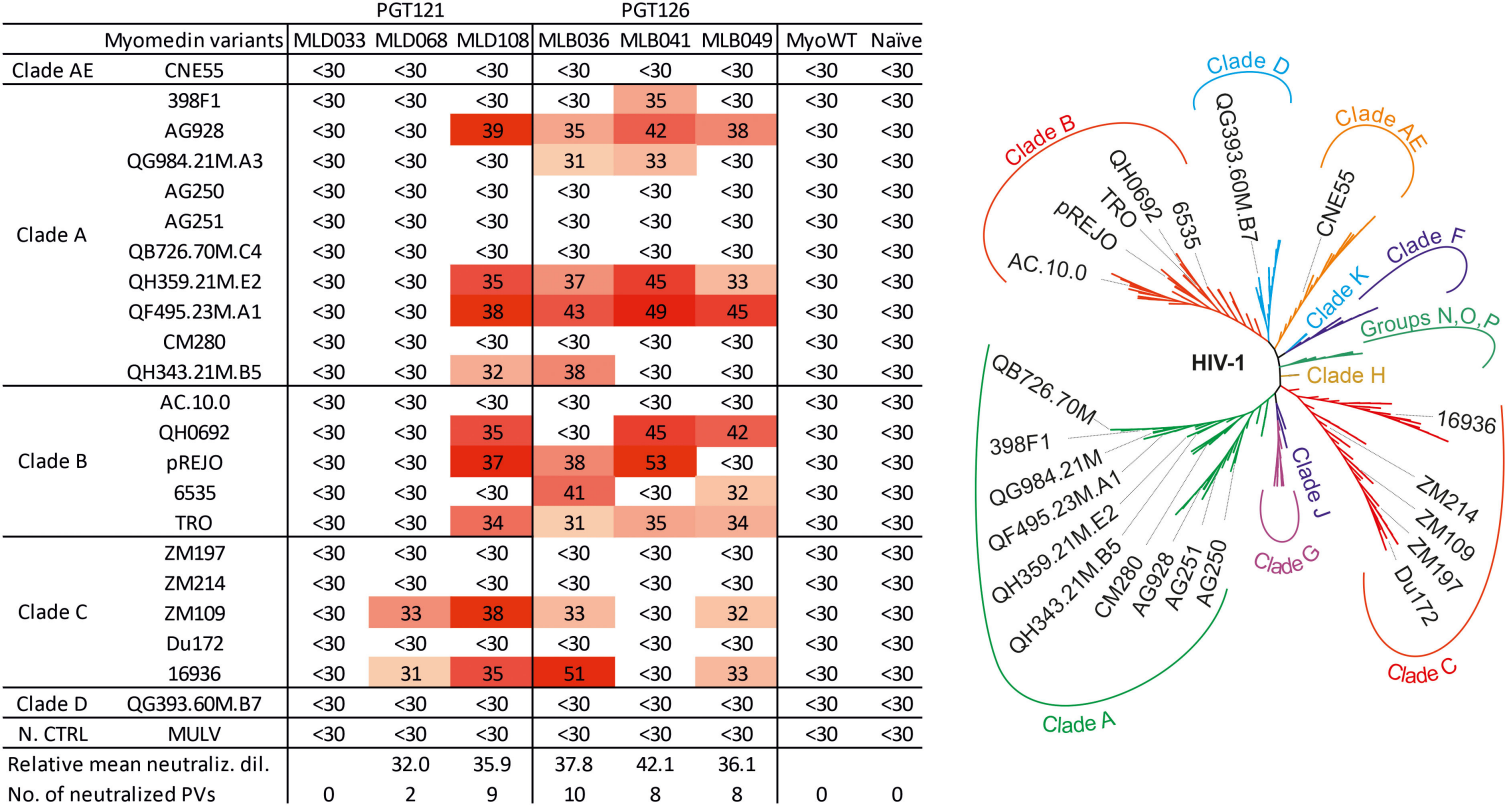
Neutralization profile of MLD and MLB protein-stimulated hyperimmune mouse sera on the panel of HIV-1 pseudoviruses. Left: 50% virus neutralization reached by reciprocal serum dilution shown by color-coded values. Relative mean neutralization dilution reflects the average values of measured titers of the used pseudoviruses. The sum of neutralized pseudoviruses per Myomedin variant-induced sera as the number of neutralized PVs is calculated at a reciprocal titer higher than 30. As a control of the neutralization assay, murine leukemia virus (MULV) was used. Right: The unrooted phylogenetic tree of the used HIV-1 pseudoviruses in this study.

To assess the neutralizing activity of serum antibodies, a panel of 22 Tier-2 pseudoviruses of Clades A, B, C, D and AE was used. **Figure 6** shows reciprocal serum dilution resulting in 50% virus neutralization, and titration curves for pseudoviruses exhibiting 50% neutralization higher than 30 are given in Suppl. **Figure 4**. Based on the percentage of neutralized pseudoviruses, best neutralizing hyperimmune mouse sera were induced by MLD108, MLB041, MLB036, and MLB049, resulting in neutralization of 10, 9, 8, and 8 out of 22 tested pseudoviruses, respectively, at dilution higher than 1:30. Among these Myomedin variants, the highest titers of binding antibodies were elicited by MLB041, but the most effective Myomedins were MLB036 and MLD108. In contrast, MLD068-immunized sera exhibited only a minimal neutralizing activity for two of the 22 tested pseudoviruses. Myomedin MLD033 failed to induce neutralizing sera in all tested mice. Ten pseudoviruses were completely resistant to all the tested hyperimmune sera (**Figure 6**). These results are in agreement with molecular modeling analysis that predicted the potential of Myomedin MLD and MLB proteins to elicit mouse antibodies neutralizing HIV-1 pseudoviruses.

## Discussion

In the crystal models for the HIV-1 Env trimer (9, 34, 40), the gp120/V3 loop is positioned at the tip of the trimer beside other gp120 (V1 and V2) variable regions. Thus, conformational changes in the Env protein during interaction with the receptor and co-receptor highly expose the most immunogenic sensitive sites, 13 amino acid-long crown, for neutralization within the V3 loop (41). In fact, a number of anti-V3 monoclonal antibodies have been isolated that incorporate gp120 glycans as part of their epitope during infection (17, 42) and after immunization (43). For instance, bNAbs, obtained from several HIV-1-infected donors demonstrated a broad spectrum of interactions with adjacent glycans on Env gp120, including N137/N156/N301 (PGT121/10-1074 family), N301 (the PGT128 bNAb), and N386/N392 (PGT135) (44). A number of these Ab, including PGT121, PGT128 and PGT135, have been demonstrated to be dependent on the intrinsic mannose N-glycan N332 residing within the intrinsic high-mannose patch, referred to as a ‘supersite’ of immune vulnerability (32, 39, 44–46). Notably, 17% of the isolated HIV-1 strains showed the switch in the N-glycosylation position from N332 to N334 site *via* double mutation, i.e., the first mutation at the 334 position to add an asparagine (N) residue followed by an introduction of Serine (S) or Threonine (T) residue at the 336 position, to produce a new NXS/T sequence motif for the N-glycosylation at the N334 site (46). Thus, HIV-1 isolates carrying N334 glycosylation site have been studied as highly resistant viral strains against a few bNAbs, especially those binding to the high-mannose patch region in gp120 protein (46, 47). Additionally, the incidence of the N295 glycan was also noted in the absence of N332 (7AA N295 N334A), which contributes to insufficient neutralization by bNAbs of the PGT128 family (48). However, some of the mannose patch-binding bNAbs have been characterized to exhibit promiscuous binding for virus neutralization by adopting distinct arrangements of N-linked glycans under the absence of the N332 glycan (46). For example, PGT130 and 74H 3L (a less altered form of PGT130) bNAbs exhibited favorable neutralization of the N334 viruses (46, 49) while PGT128 demonstrated neutralization of only N334 viral strains in the presence of the N295 glycan and all gp120/V3 (i.e., 7AA N334 N295 variant) (48). Similarly, PGT126 was also demonstrated for binding to the entire V3 loop glycopeptides decorated with high-mannose glycans but not to the glycopeptides displaying the complex-type N-glycan (50). These reports further support the results that PGT128 and PGT126 promiscuously bind to the high mannose glycans in the context of the V3 loop peptide, in agreement with previously documented mutational analysis (45).

Recently, crystal structure analysis of HIV-1 constrained gp140 trimer exhibited a tight association between V1V2 and V3 regions. Thus, V1V2 seems to restrict access to the V3 region while securing itself by N-glycosylation (9). Moreover, V1V2 and V3 restricted the access to CD4bs by glycans *via* a shielding effect (51). Importantly, deletion of the highly conserved glycan at the N301 site has been demonstrated to reveal V3 loop and CD4 binding site epitopes (46, 52, 53). However, Moyo et al. (54) reported a subtype C strain, named CAP45.2.00.G3, in which the elimination of glycan at the N301 site did not cause an increase in sensitivity to a substantial quantity of sera (61/64 panels) from chronically infected patients (54). A vital role of the glycan at the N301 position in protecting other strains from neutralization was reported (53, 54). Goo et al. and Sok et al. established that some viral strains carrying essential N301 and N332 V3 glycans can be resistant to neutralization by the potent bNAbs, PGT121 and PGT128, due to unknown mechanisms that resulted in altered Env conformation (46, 55). Based on the published data, we propose that Myomedin scaffold variants mimicking the distinct glycan epitopes within high mannose patches of the gp120 protein should be beneficial for protecting against HIV infection. Based on the structural data, we assembled models of HIV-1 Env in complex with bNAbs PGT121 or PGT128. In these complexes, we identified antibody interaction residues and annotated them formally by corresponding Env counterparts. In the case of PGT121, four Env regions were designated to be in a close proximity to antibody residues (non-hydrogen atom distance within 5 Å, for more details, see **Supplementary Figure S2**). One of them, N137 glycan is recognized by residues Y33, Y50, S54, G55, D56, N58, H97, R99, W100J, F100K and T100L of IgG heavy chain, and residues W91, D92 and S93 of the IgG light chain. The second region is formed by N156 glycan recognized by E25 of the IgG light chain. The third one is represented by N332 glycan recognized by a stretch of residues H97, G98, R99, R100, I100A, Y100B, G100C, I100D and V100E of the IgG heavy chain, and IgG light chain residues S30, R31, N50, N51, Q52, D53 and D66A. The last region is defined by direct protein-protein interactions between Env amino acid residues V134, T135, N136, N137, I323, G324, D325, I326, R327, Q328, H330, T415, L416, P417, and PGT121 heavy chain residues Y100B, G100C, I100D, A100F, F100G, N100H and E100I, and light chain residues L28, G29, S30, F67, S93 and R94.

The binding of Myomedin variants MLD033, MLD068 and MLD108 to PGT121 predicted by docking presented in **Figure 3** is utilizing antibody residues involved in recognition of all Env regions (**Supplementary Figure 2**). This is in agreement with the experimentally observed mimicking potential of all the three MLD variants. Interestingly, MLD033 did not exhibit any neutralizing potential in the panel of 22 tested pseudoviruses, and MLD068 was able to suppress pseudoviruses only in two cases of Clade C. On the other hand, MLD108 neutralizes a broader range of pseudoviruses across all clades (**Figure 6**). Results presented in **Supplementary Figure 2** suggest that MLD108 differs from the other two MLD variants by a higher number of close protein-protein interactions that would be otherwise shielded by surrounding glycans. This may result in higher immunogenicity leading to the neutralization of 9 from 22 tested pseudoviruses.

In the case of the PGT128, four close interacting regions were identified. The first one containing the glycan N137 is recognized by IgG heavy chain amino acid residue E98 and IgG light chain N31. The second region is defined by N301 glycan and antibody residues S28, A30, A31, C32, H52A, C52B, A52C, S52D, Y52E, L71, T73 and P74. The third one is formed by N332 glycan and IgG heavy chain antibody residues W52F, N53, G55, W56, T57, Y58, H59, N60, P61, K64 and W100E, and IgG light chain residues V92, G93, N94, W95 and D95A. The last, protein-protein region is characterized by Env residues N137, T297, R299, N301, D321A, I322, I323, G324, D325, I326, R327, V442 and R444, IgG heavy chain antibody residues C32, A52C, S52D, Y52E, Y52E, E98, L100, R100A, Y00B, T100C and D100D, and IgG light chain residue N31 (**Supplementary Figure 3**).

As predicted by docking, all Myomedins MLB036, MLB041 and MLB049 interact with all four specified PGT126 regions. This correlates with experimental data from competition ELISA supporting a mimicking potential for all MLB binders. Patterns of most probable interactions for MLB036 and MLB049 overlap in all four regions (**Supplementary Figure 3**). In contrast, MLB041 differs in binding to N301, N332 and protein-protein regions by more close contact with the N332 region. This observation could be associated with the better neutralizing potency of MLB041 seen for a total of eight pseudoviruses from Clade A and B (**Figure 6**). It has also been described that N332 glycans are significantly underrepresented in Clade C viruses (47). Myomedins MLB036 and MLB049 neutralize a similar set of pseudoviruses across the clades with a breadth of 10 of 22 and 8 of 22, respectively.

To interpret the neutralization efficacy of hyperimmune sera on the panel of selected pseudoviruses, we compiled Env protein sequences for each of the 22 chosen pseudoviruses into the table (**Supplementary Table 1**) and analyzed the occurrence of mutations in consensus glycosylation sites together with sequence variability in V1/V2 and V3 hypervariable loops. The results revealed that all 22 variants carry WT N301 glycan amenable for the neutralization by bNAbs of the PGT series. It has been described that loss of N332 glycan is associated with complete resistance to PGT121 (56). As expected, eight viruses with WT N332 glycan were neutralized. Glycan rearrangement by a shift from N332 to N334 or back was described for several viruses (47). In our experiments, five pseudoviruses carry N332 mutation compensated by S334N glycosylation, and four of them were neutralized. Because of a critical N332 glycan loss, two other viruses without compensated N332 mutation were not neutralized. In two pseudoviruses, mutation D325N as a part of the conserved GDIR V3 loop sequence leads to substantial suppression of neutralization. The remaining 10 pseudoviruses were not neutralized, most probably due to V1V2 loop variability affecting N137 glycan and its neighbourhood. In summary, a total of 12 from 22 viruses (54%) were neutralized (**Supplementary Table 1**). This observed virus-neutralizing coverage of MLB/MLD can be compared with that published for PGT121 and PGT126 bNAbs. Walker et al. (23) reported that PGT121 in the highest concentration range (1-50 µg ml^-1^) neutralized 70% of viruses, while the middle range concentration (0.1-1 µg ml^-1^) neutralized 57% of viruses and the lowest one (≤ 0.1 µg ml^-1^) only 44% viruses. Compared with this published coverage, MLD108-elicited sera neutralized 9 of 22 pseudoviruses (40.90%). Similarly, PGT126 in the concentration range of 1-50 µg ml^-1^ neutralized 60% of viruses, 0.1-1 µg ml^-1^ neutralized 50% of viruses and below 0.1 µg ml^-1^ neutralized only 40% of viruses (23). In this context, MLB036-stimulated sera reach 45.45% neutralizing activity (10 of 22 pseudoviruses).

The comparison of sequence similarity of MLB and MLD proteins regarding the neutralizing potential reveals several interesting findings. First, MLB036 protein shares a loop L2 sequence with the parental non-randomized Myomedin, which seems to not significantly affect their epitope mimicking and neutralizing potential compared to MLB049. The L2 loop-inherited sequence can probably still mimic the glycan due to the polar amino acid residues present in the RNT sequence. Second, five amino acid residues of the L3 loop (IVTPL) share sequence identity between MLB041 and MLB049, but their neutralizing potential found in hyperimmune murine sera mainly differs in Clade A, B and C pseudoviruses (**Figure 6**). This suggests that a complex of interacting residues involving the L1 and L2 amino acids must direct the particular binding mode and predicted orientation. Finally, neutralizing potential of MLD108-stimulated murine sera with those elicited by MLB036 and MLB049 is strikingly similar, yet MLD and MLB proteins were selected independently from different collections of cDNA libraries as a result of targeting different monoclonal antibodies. These findings showed that if paratopes of the original bANbs have overlapping binding sites involving the same V3 glycans, both MLD108 (ligand of PGT121) and MLD036 with MLB049 (ligands of PGT126) must elicit a similar set of neutralizing antibodies able to block almost the identical pseudoviral portfolio of gp120 epitopes (**Figure 6**). This could be another valuable feature of Myomedin antigens in HIV vaccine development.

We realize that Ab is not intrinsically specific for a single epitope and it could have been elicited by any one of the antigens with which particular Ab is able to react (57). Thus, our strategy is based on the *in vitro* identification of proteins binding selectively bNAb from highly complex combinatorial library to identify a set of binders for the selected bNAb. Binders were subsequently tested in experimental animals as the antigens for elicitation of gp120 binding and HIV-1 pseudoviruses neutralizing sera. Although we opted capturing the tested mAb on ELISA plates during binders' identification, we were aware of potential conformational changes in the bound mAb. Therefore, we used several competition assays to test the mAb-to-binder, Ab-to-gp120, and, after immunization, also hyperimmune serum-to-binder and serum-to-gp120 interaction in soluble conditions, which is important for antibody interaction with its target. Because during the neutralization process *in vivo* the target Env protein is a part of virus membrane, final analysis was focused on testing the sera neutralizing properties utilizing HIV-1-pseudotyped virus assay. Finally, we performed structural modeling of the interaction of best *in vitro* and *in vivo* acting binders with mAb using available crystal structures to, at least partially, address the nature of binders to mAb interaction. Our “non cognate antigen” approach is an alternative to strategies of structure-based reverse vaccinology (SBRV) identifying gp120-derived antigen for immunization by detail analyses of the pair mAb-recombinant gp120 (gp140, SOSIP etc) which did not succeed to provide a construct for the generation of successful broadly effective preventative vaccine. The aspects potentially associated with the failure of SBRV are discussed in detail by van Regenmortel (57).

The prominence of stimulating a diverse array of antibodies targeting distinct epitopes for protection against HIV has been noted in several studies (58). Neutralizing antibodies against V3 or other sites, such as the CD4-binding site, are instantly selected for neutralization-resistant variants (59). However, the combination of monoclonal antibodies was required to overcome viremia after infection was established (60). Intriguingly, the endogenous induction of antibodies against the V3 crown after passive transfer with bNAbs was found beneficial as the different antibodies can work in synergy against virus escape (61). In this context, several collections of non-cognate ligands targeted to paratopes of prominent HIV-1 bNAbs, such as VRC01, 10E8, and PGT121/PGT126, specific for antigens within the CD4 binding region, gp41 MPER and gp120 V3 loop, using a combinatory immunization approach consisting of the best VRA, MLA and MLB/MLD variants may provide an opportunity to fight against viral mutations allowing escape from host immune surveillance and significantly widen the neutralizing coverage, leading to extension of anti-viral protection. Glycan mimicking Myomedins described in this work will play an essential role in this effort. Considering the negative controls, moderate HIV-1-neutralizing titers detected in murine hyperimmune sera in combination with a broad coverage of the tested pseudoviruses are encouraging for further studies. These will be focused on the optimization of immunization protocol to achieve higher neutralization titers, for example by testing various adjuvants, by multimerizing of the selected Myomedin variants for effective crosslinking of specific B cell receptors (BCRs), or by coupling the Myomedins with carrier proteins.

## Data availability statement

The datasets presented in this study can be found in online repositories. The names of the repository/repositories and accession number(s) can be found in the article/**Supplementary Material**.

## Ethics statement

The animal study was reviewed and approved by The Ethics Committee of the Faculty of Medicine and Dentistry (Palacky University, Olomouc, Czech Republic) and the Ministry of Education, Youth and Sports, Czech Republic (MSMT-9487/2019-3).

## Author contributions

PM and MR conceived and designed the study. VDL, MK, and TZ performed a large-scale screening of Myomedin library and selection of MLB and MLD variants. HP, JŠ, and MM performed protein characterization assays. JČ performed *in silico* modeling of Myomedin proteins interactions. PK and MKr performed the immunological analyses. PK, LRK, and MR performed immunization experiments. EV and LRK engineered and isolated recombinant gp120 proteins and isolated plasmids for pseudoviruses preparation. PK performed virus-neutralizing assays. PM and MR supervised the study. PM, MR, JČ, PK, and SB wrote the manuscript with input from all authors. All authors have read and agreed to publish this version of the manuscript.
