# Supplementary material for: Myomedin replicas of gp120 V3 loop glycan epitopes recognized by PGT121 and PGT126 antibodies as non-cognate antigens for stimulation of HIV-1 broadly neutralizing antibodies
Source: Front Immunol. 2022 Dec 8;13:1066361. doi: 10.3389/fimmu.2022.1066361 (PMC9772448; doi:10.3389/fimmu.2022.1066361)
Supplement: Supplementary file 1 [file DataSheet_1.docx]

Supplementary information

**Myomedin replicas of gp120 V3 loop glycan epitopes recognized by PGT121 and PGT126 antibodies as non-cognate antigens for stimulation of HIV-1 broadly neutralizing antibodies**

Veronika Daniel Lišková, Petr Kosztyu, Milan Kuchař, Jiří Černý, Shiv Bharadwaj, Hana Petroková, Eliška Vróblová, Michal Křupka, Michal Malý, Tereza Zosinčuková, Josef Šulc, Leona Rašková Kafková, Milan Raška, Petr Malý.

**
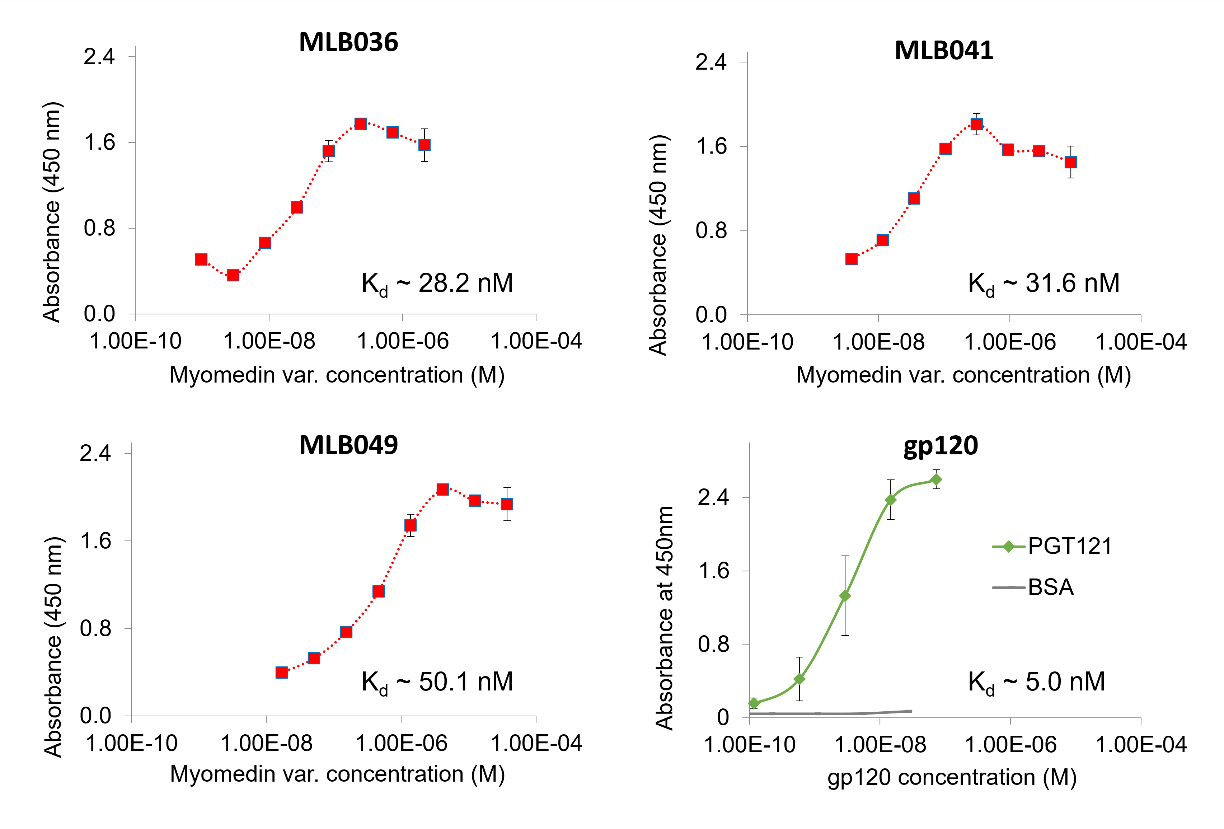
**

**Suppl. Figure 1. Binding of MLB protein variants to PGT126 bnAb in ELISA.**The protein variants MLB (His_6_-Myo-V5) were produced in *E. coli* BL21 strain, protein was purified using Ni-NTA agarose and assayed in ELISA. The binding to PGT126 bnAb was visualized by the anti-V5 antibody conjugated with HRP. gp120 binding to PGT121 is also demonstrated. Each point is shown as the mean value of triplicates with standard deviation.

**Suppl. Figure 2.** **Interatomic distances within 6 Å between residues of PGT121 bNAb and particular Myomedin MLD variants.** The color-coded numbers indicate the distance of particular atoms in the deposited crystal structure and in model complexes. See the file: PGT121_contacts.pdf

Link: <https://zenodo.org/record/6913156/files/PGT121_contacts.pdf?download=1>

**Suppl. Figure 3.** **Interatomic distances within 6 Å between residues of PGT126 bNAb and particular Myomedin MLB variants.** The color-coded numbers indicate the distance of particular atoms in PGT126 homology model and in model complexes. See the file: PGT126_contacts.pdf

Link: <https://zenodo.org/record/6913156/files/PGT126_contacts.pdf?download=1>

**Suppl. Table 1.** Overview of mutations in particular glycan sites for all 22 pseudo viruses used in this study in combination with observed neutralization pattern of hyperimmune mouse sera induced by MLD and MLB mimotopes. See the file: Clades and Predicted_sites_2022-08-04-1.pdf

Link: <https://zenodo.org/record/6913156/files/Clades%20and%20Predicted_sites_2022-08-04.pdf?download=1>


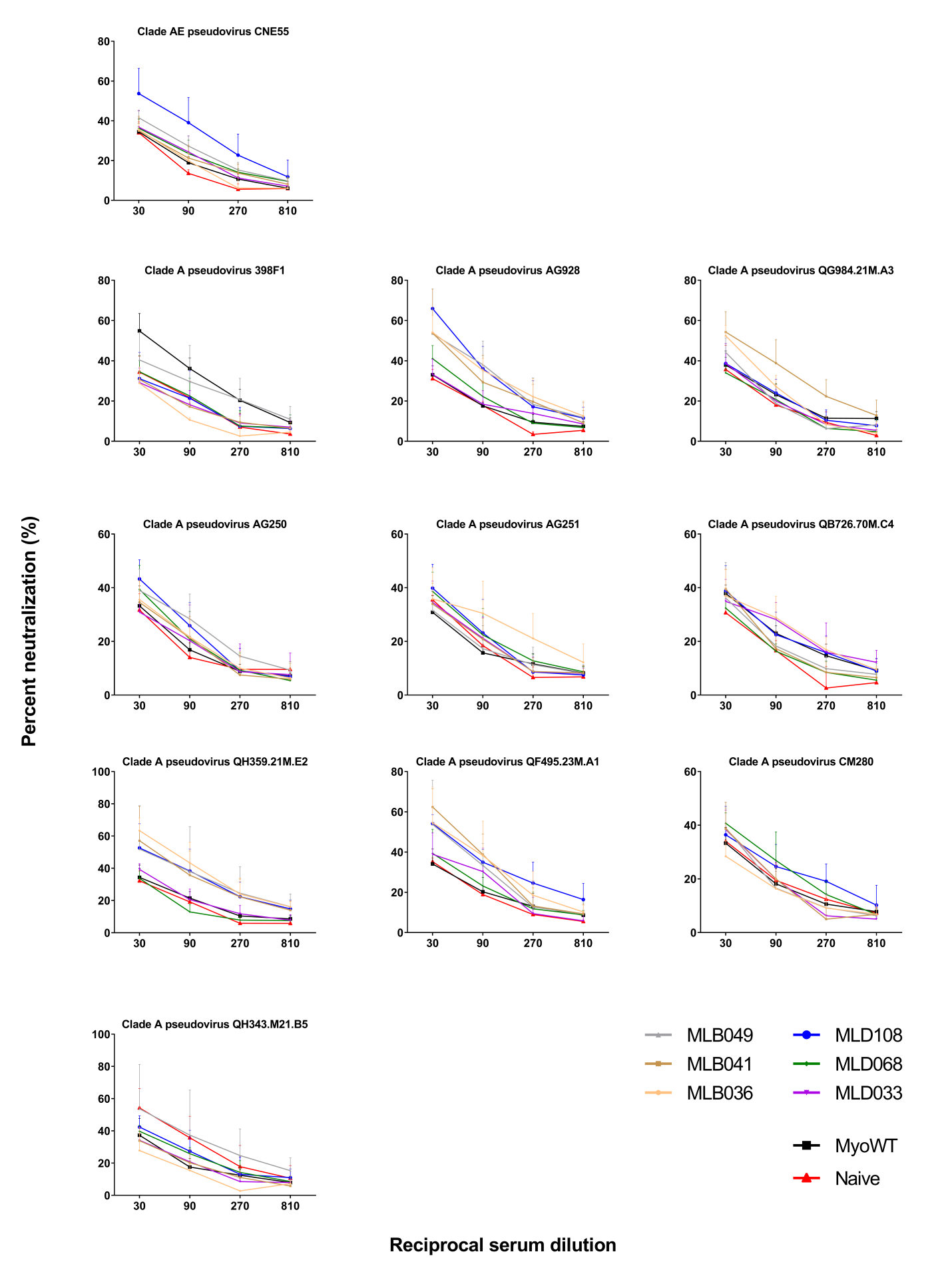


**
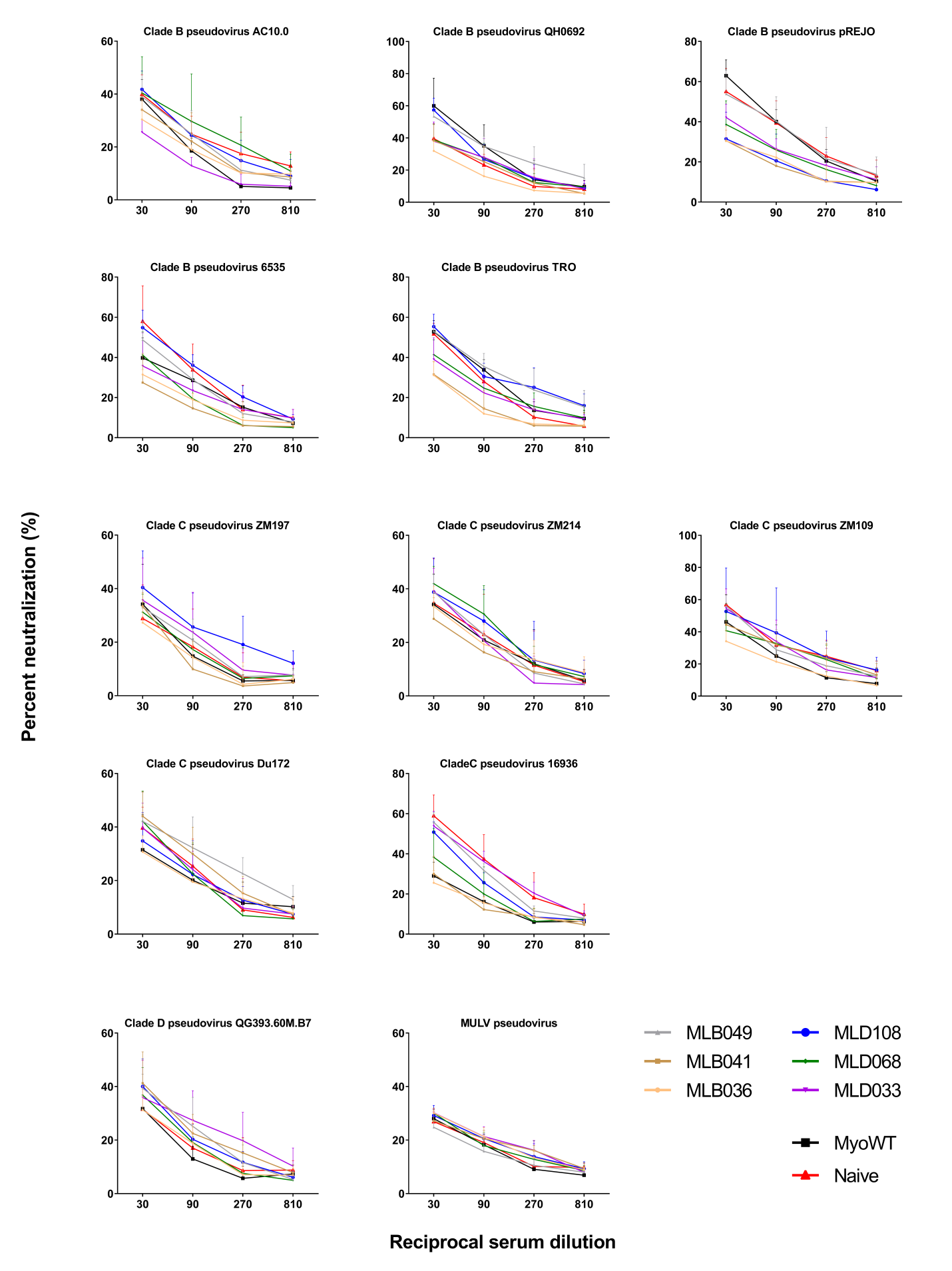
Suppl. Figure 4:** **Neutralization titration of sera from for MLB- and MLD-immunized mice.** Mice were immunized by the intradermal administration of four doses of individual MLB or MLD variants including wild-type (MyoWT). The order of immunization was detailed in Fig. 4A. Each group consisted of five animals. Neutralization assays were performed using a set of HIV-1 Clade A, B, C, AE, and D pseudoviruses of Tier 2 or 3 with TZM-bl indicator cells. Serially diluted serum samples in duplicates were incubated with pseudoviruses. The pseudoviruses load was set to achieve approximately 150 000 RLU in 150 µl of DMEM in the absence of sera. After the incubation pseudovirus with serum, 10^4^ TZM-bl cells were added, incubated, lysed, and after addition of substrate, luminescence was measured.
